# Knowledge and Attitude of General People Towards Symptoms of Heart Attack and the Impact of Delay Time in Riyadh, Saudi Arabia

**DOI:** 10.7759/cureus.32758

**Published:** 2022-12-20

**Authors:** Khalid M Al Harbi, Waleed A Alluhidan, Malek I Almatroudi, Naif I Almuhanna, Naif M Alotaibi

**Affiliations:** 1 Department of Internal Medicine, Imam Mohammad Ibn Saud Islamic University, Riyadh, SAU; 2 College of Medicine, Imam Mohammad Ibn Saud Islamic University, Riyadh, SAU

**Keywords:** cvd risk factors, hypertension, cardiovascular disease (cvd), acute coronary syndrome (acs) and stemi, awareness of cardiovascular disease

## Abstract

Background: Cardiovascular disease is prevalent worldwide. The goal of this research is to evaluate the knowledge of Riyadh, Saudi Arabia, population about heart attack symptoms and risk factors.

Methodology: A one-year cross-sectional study was carried out. The study was conducted on 385 individuals in Riyadh, Saudi Arabia. We used the Acute Coronary Syndrome Response Index, with additional questions added, such as risk factors of heart attack and physical activity time. An anonymous self-administered online questionnaire was used to collect the data.

Results: We collected data from 440 participants, but only 385 were included in the analysis. Males represented 41.4% of the participants. In terms of participant knowledge of heart attack symptoms, we found that chest pain or pressure was the most common (80.5%), followed by shortness of breath (77%) and weakness and fatigue (72.0%). In addition, 90.2% and 90.7% of the participants knew that smoking and obesity were risk factors for heart attacks. Furthermore, 46% of participants said they “would not be at all certain” of identifying the symptoms and indicators of a heart attack in another person and 45.7% “in themselves.” We found that males were more likely than females to have low knowledge (RR: 1.84, 95% CI: 1.24:2.72, *P *= 0.002).

Conclusion: Our findings suggest that there is a lack of awareness of the heart attack warning signs and symptoms. We propose that future local campaigns focus on increasing awareness and recognition of heart attack symptoms.

## Introduction

Cardiovascular disease (CVD) is responsible for nearly 40% of mortalities in the European Union [1] and 35% in the United States [2]. The most common manifestation of CVD is an acute coronary syndrome (ACS), which referred to a group of disorders related to acute myocardial ischemia (AMI) resulting from a sudden decrease in coronary blood flow [2]. Diabetes, hypertension, dyslipidemia, obesity, smoking, poor dietary habits, and an inactive lifestyle are all risk factors for ACS [3]. In 2010, ACS was responsible for approximately 11.75% of all deaths in the Kingdom of Saudi Arabia [4]. Moreover, dyslipidemia and hypertension are the most common risk factors. According to a recent study, 15% of the total population has one cardiovascular risk factor, 24.0% has two risk factors, 16.9% has three risk factors, and 17.6% has four risk factors. [5]. In KSA, a study found that approximately 40% of STEMI patients failed to achieve a door-to-balloon time of 90 minutes, with women being less likely to achieve this time, and that the in-hospital deaths were 4% [6].

Knowing the major symptoms of a cardiovascular emergency can save lives by allowing bystanders and patients to respond quickly. According to Alfasfos et al.’s study on Jordanians, the most common ACS symptom, according to the most of responses, is chest pain, followed by arm numbness and shortness of breath, while the majority missed symptoms like abdominal pain and heartburn/indigestion [7]. In another study, Noureddine et al. conducted on Lebanese people, the majority of participants (>85%) recognized typical myocardial infarction (MI) symptoms such as chest pain and sweating, but fewer recognized dyspnea (66%), arm pain (62%), and nausea/vomiting (52%). Less than half of the participants reported atypical symptoms such as heartburn and jaw pain. [8]. These are the only studies that have been conducted on Arab countries. According to Fang et al., in a study done in the United States, the adjusted percentage of people who were aware of all five common heart attack symptoms (jaw, neck, or back pain; weakness or lightheadedness; chest pain; arm or shoulder pain; and shortness of breath) increased from 39.6% in 2008 to 50.0% in 2014 and 50.2% in 2017. [9]. Similar studies have been conducted in Beijing and Shanghai, China, and Bangalore, India. Researchers discovered that only 60% of people in all cities reported that chest pain/discomfort is a sign of AMI. In Bangalore, however, 21% of respondents could not name a single symptom [10].

The only factor influencing treatment outcome is timely arrival at the hospital. The amount of time that passed between the onset of symptoms and the arrival of medical services for treatment is referred to as patient delay; understanding delay time and its impact on the outcome is crucial for all patients. Every 30 minutes of delay raises one-year mortality by 7.5%, emphasizing the importance of early symptom recognition and appropriate timely response [11]. According to McGinn et al., a four-hour delay reduced the likelihood of receiving thrombolytic therapy or percutaneous coronary intervention and increased the likelihood of death from non-ST-elevation myocardial infarction (NSTEMI), heart failure, or shock [12]. Patients delay due to ignorance or the fear that their complaints will annoy others. According to Moser et al., the reasons for the delay did not differ significantly between men and women, but a prevailing factor in women’s delay was not wanting to bother others, whereas ignorance about thrombolytic therapy was a prevailing factor in men’s delay [13]. According to Moser et al., if reperfusion occurs within one hour of symptom onset, survival rates improve by 50% and by 23% if it occurs within three hours of symptom onset. [14]. Patients’ delays go beyond a lack of knowledge because many studies show that despite patients’ knowledge about MI symptoms or having a history of MI, the delay still occurs, implying that patients’ delays are caused by a variety of factors: cognitive, social, and emotional factors need to be investigated further to gain a better understanding of the interconnected nature of patients’ delay [15].

Multiple studies have shown that women typically delay longer than men: for example, Lichtman et al. found that young women (<55 years old) delayed longer than older patients. This delay resulted in a higher mortality rate in young women due to MI, with contributing factors including (1) the nature and duration of prodromal symptoms varied significantly; (2) patients who miscalculated their personal risk of heart disease and frequently misattributed symptoms to noncardiac causes; (3) competing and conflicting priorities that influenced decisions to seek medical care; (4) the healthcare system's failure to respond consistently to young women with MI, resulting in the workup and diagnosis delays; and (5) participants who did not seek primary care regularly, including cardiovascular disease prevention [16]. All of the preceding examples help to explain why patients delay seeking medical care; some of the major reasons include not understanding their presentation and its significance, attributing symptoms to another illness, having conflicting interests, and not wanting to bother others. The general public’s awareness of MI presentation could play a substantial role in reducing delay time, thus significantly lowering the one-year mortality rate. Another important aspect of raising MI awareness is knowledge of thrombolytic therapy. [9]. These percentages are concerning because, despite advances in medical knowledge over the last 20 years, public knowledge and awareness of AMI are lower than we would like.

Our goal in this study is to assess the knowledge of the Riyadh (capital of Saudi Arabia) population about the early symptoms of ACS and the risk factors that can lead to ACS. Knowing the knowledge level can help us to reduce the burden of the disease, as well as the mortality rate, and prevent the risk factor by educating people more about them and encouraging a healthy lifestyle, as well as giving an idea about the common presentation for patients with cardiac events and the impact of delay time on the patients.

## Materials and methods

Study design, area, and settings

From July 2021 to July 2022, a cross-sectional study of 385 people was conducted in Riyadh, Saudi Arabia.

Measures

The questionnaire was divided into two parts. The first part includes socio-demographic information. In the second part, we used the ACS Response Index by Barbara Riegel et al. [17], which consists of 33 questions graded on different scales. The first scale measures knowledge of heart attack symptoms and consists of 21 questions with a 0,1 scale (0 means no, 1 yes). The second scale measures attitudes toward heart attacks by asking five questions on a four-point scale (1: not at all; 2: quite sure; 3: pretty sure; 4: very sure). The third scale measures beliefs by asking seven questions on a four-point scale (1: strongly agree; 2: agree; 3: disagree strongly; 4: disagree). Additional questions, such as knowledge of the risk factors of heart attack and physical activity, were added. The survey was widely disseminated via social media. If any questions were unclear to the participants, they were given an e-mail address to contact the research team, and all inquiries and questions about the survey were answered. Convenience sampling was the sampling technique used. Moreover, a bilingual translator translated the questions from English to Arabic. We ensured that every participant’s privacy was protected. This study followed the Helsinki 2013 principles. In the analysis, only completed questionnaires with informed consent were included, and incomplete ones were discarded.

Data collection

An anonymous self-administered online questionnaire was used to collect data from January 2022 to April 2022. The questionnaire form included questions about the symptoms, attitudes, and beliefs of the participants toward MI. 

Data analysis

A biostatistician examined the data. Continuous variables were presented as means and standard deviations, while categorical variables were presented as frequencies and percentages. P-value was considered significant if ≤0.05.

Sample size

Riyadh population: Approximately 8 million. Sample size: 385 completed questionnaires. Confidence level: 95%. The margin of error is 5%.

Inclusion criteria

Adults older than 18 years living in Riyadh, Saudi Arabia. English or Arabic speakers.

Exclusion criteria

Adults with mental illnesses, individuals younger than 18 years or people living outside of Riyadh, non-Arabic or English speakers.

Ethical consideration

This study was carried out with Imam Mohammad Ibn Saud Islamic University IRB approval, and only data matching the objectives of the study will be used. Participants' data and names that do not match the objectives of the study will not be collected and will not be used. After explaining the study’s objectives and goals, all participants were required to provide permission to take part in this research.

## Results

We were able to collect data in this study from 440 participants. However, only 385 were included in the analysis (Table 1).

**Table 1:** General characteristics of participants

		Count	%
Gender	Male	182	41.4%
	Female	258	58.6%
Marital status	Single	145	33.0%
	Married	279	63.4%
	Divorced	16	3.6%
Age	18-23	78	17.7%
	24-29	75	17.0%
	30-35	54	12.3%
	36-41	63	14.3%
	42-47	72	16.4%
	48-53	52	11.8%
	54-59	27	6.1%
	60-65	16	3.6%
	66-71	2	0.5%
	71 or older	1	0.2%
Education	Less than secondary school	14	3.2%
	Secondary school	82	18.6%
	Bachelor	284	64.5%
	Master	49	11.1%
	Doctorate	11	2.5%
Nationality	Saudi	399	90.7%
	Non-Saudi	41	9.3%

Males made up 41.4% of the participants. The majority of the participants were younger than 41 years (61.3%), 17.7% of them ages were between 18 and 23, and 16.4% were between the ages of 42 and 47. Moreover, 64.5% of the respondents reported having a bachelor’s degree, while 18.6% had a secondary education.

Table 2 discusses the knowledge of the participants about the symptoms of a heart attack.

**Table 2:** Knowledge of the participants toward the symptoms of the Heart attack

	No		Yes	
	Count	Row N %	Count	Row N %
Lower abdominal pain	422	95.9%	18	4.1%
Arm pain or shoulder pain	139	31.6%	301	68.4%
Arm paralysis	215	48.9%	225	51.1%
Back pain	328	74.5%	112	25.5%
Chest pain/pressure	86	19.5%	354	80.5%
Chest discomfort (heaviness, burning)	145	33.0%	295	67.0%
Cough	301	68.4%	139	31.6%
Dizziness, lightheadedness	217	49.3%	223	50.7%
Headache	291	66.1%	149	33.9%
Heartburn/stomach problem	378	85.9%	62	14.1%
Jaw pain	359	81.6%	81	18.4%
Loss of consciousness	176	40.0%	264	60.0%
Nausea/vomiting	289	65.7%	151	34.3%
Neck pain	295	67.0%	145	33.0%
Numbness/tingling in arm or hand	136	30.9%	304	69.1%
Pale, change of color	221	50.2%	219	49.8%
Palpitations/rapid heart rate	131	29.8%	309	70.2%
Shortness of breath	101	23.0%	339	77.0%
Slurred speech	164	37.3%	276	62.7%
Sweating	152	34.5%	288	65.5%
Weakness/fatigue	123	28.0%	317	72.0%

The most common symptoms reported by participants were chest pain or pressure (80.5%), followed by shortness of breath (77%), weakness and fatigue (72.0%), palpitations and rapid heart rate (70.2%), numbness or tingling in arm and hand (69.1%), and arm pain or shoulder pain (68.4%). On the other hand, most of the participants reported that lower abdominal pain, heartburn or stomach problem, and jaw pain (95.9%, 85.9%, and 81.6%, respectively) were not symptoms of a heart attack.

Table 3 shows the knowledge level of participants for coronary artery disease.

**Table 3:** Knowledge of risk factors for coronary artery disease

	No		Yes		I do not know	
	N	N %	N	N %	N	N %
Do you think smoking increases your risk of getting Myocardial infarction	6	1.4%	397	90.2%	37	8.4%
Do you think Hypertension increases your risk of getting Myocardial infarction	17	3.9%	383	87.0%	40	9.1%
Do you think high levels of Cholesterol increases your risk of getting Myocardial infarction	19	4.3%	390	88.6%	31	7.0%
Do you think Obesity increases your risk of getting Myocardial infarction	19	4.3%	399	90.7%	22	5.0%
Do you think diabetes is a risk factor for coronary heart disease	128	29.1%	210	47.7%	102	23.2%
Do you think family history of coronary heart disease may affect the risk of getting Myocardial infarction	90	20.5%	273	62.0%	77	17.5%
Do you think having a previous coronary artery disease may increase the risk of getting	7	1.6%	362	82.3%	71	16.1%
Do you think it is possible to lower the risk of coronary artery disease for elderly people	31	7.0%	314	71.4%	95	21.6%

We found that 90.2% of participants were aware that smoking could increase the risk of having a myocardial infarction (MI), while 90.7% were aware that obesity is a risk factor for MI. Furthermore, 88.6% and 87.0% knew that high levels of cholesterol and hypertension are other risk factors for MI, respectively. On the other hand, only 47.7% of participants knew that diabetes mellitus is a risk factor for MI.

**Figure 1:**
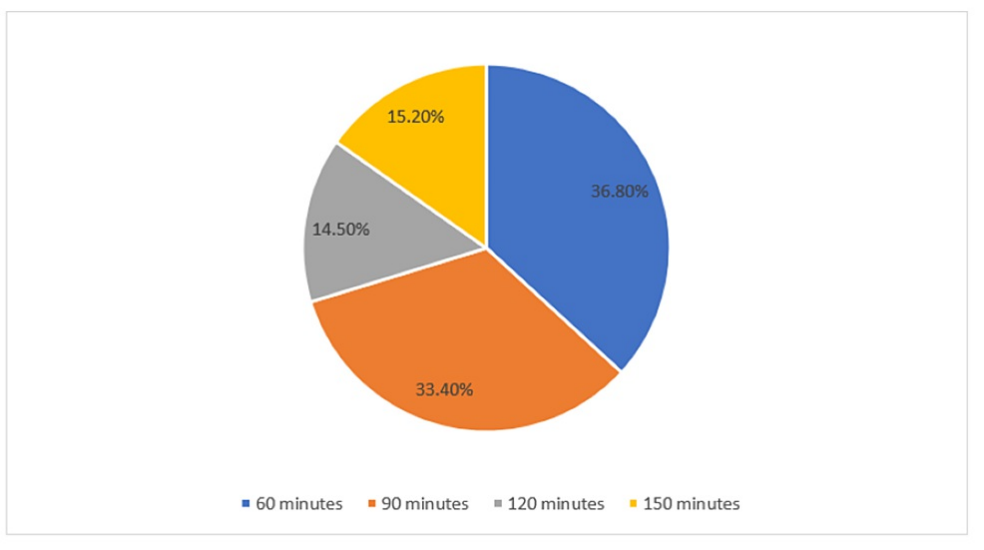
How much do you think the minimum moderate-intensity physical activity per week is required?

We found that 36.8% reported that 60 minutes of moderate-intensity physical activity per week is required to reduce the risk of MI, while 33.4% reported the need for 90 minutes (Figure 1).

Attitudes and beliefs of the participants towards MI are shown in Table 4.

**Table 4:** The attitudes and beliefs of the participants toward MI

	Strongly disagree		Disagree		Neutral		Agree		Strongly agree	
	N	%	N	%	N	%	N	%	N	%
If I have chest pain that doesn’t stop after 15 minutes, I should get to the hospital as soon as possible	5	1.1%	73	16.6%	0	0.0%	161	36.6%	201	45.7%
I would be embarrassed to go to the hospital if I thought I was having a heart attack but I wasn’t	144	34.3%	199	47.4%	0	0.0%	52	12.4%	25	6.0%
If I thought I was having a heart attack, I would wait until I was very sure before going to the hospital	151	34.3%	190	43.2%	3	0.7%	68	15.5%	28	6.4%
If I thought I was having a heart attack, I would rather have someone drive me to the hospital than have an ambulance come to my home	72	16.4%	136	30.9%	7	1.6%	129	29.3%	96	21.8%
Because of the cost of medical care, I would want to be absolutely sure I was having a heart attack before going to the hospital	156	37.1%	174	41.4%	0	0.0%	58	13.8%	32	7.6%
If I’m having chest pain and I’m not very sure if it’s a heart attack, I should go to the hospital	14	3.3%	93	22.1%	0	0.0%	188	44.8%	125	29.8%
If I thought I was having a heart attack, I would go to the hospital right away	12	2.9%	21	5.0%	0	0.0%	105	25.0%	282	67.1%

Regarding the attitudes and beliefs of the participants toward MI, we discovered that the majority of participants will go to the hospital if they experience chest pain that does not go away after 15 minutes. (45.7% strongly agreed, and 36.6% agreed). In addition, 47.4% of participants disagreed, whereas 34.3% strongly disagreed with the statement that they would be embarrassed to go to the hospital if they were not suffering from a heart attack. Furthermore, most of the participants would not go to the hospital unless they were certain. Moreover, 30.9% disagreed, and 16.4% strongly disagreed that if they had symptoms of a heart attack, they would like someone to drive them to the hospital rather than having an ambulance come to their home; yet 29.3% and 21.8% agreed and strongly agreed with this statement. Most of the participants stated that the cost of medical care would not influence their decision to go to the hospital if they suspected they experienced a heart attack. Lastly, 67.1% of participants strongly agreed that they would go to the hospital immediately if they suspected they were having a heart attack.

Table 5 shows the attitude of the participants in recognizing the symptoms of a heart attack.

**Table 5:** Attitude of the participants in recognizing the symptoms of a heart attack

	Not at all sure		Little sure		Pretty sure		Very sure	
	Count	%	Count	%	Count	%	Count	%
How sure are you that you could recognize the signs and symptoms of a heart attack in someone else?	193	46.0%	164	39.0%	45	10.7%	18	4.3%
How sure are you that you could recognize the signs and symptoms of a heart attack in yourself?	192	45.7%	150	35.7%	62	14.8%	16	3.8%
How sure are you that you could tell the difference between the signs or symptoms of a heart attack and other medical problems?	253	60.2%	125	29.8%	30	7.1%	12	2.9%
How sure are you that you could get help for someone if you thought they were having a heart attack?	256	61.0%	97	23.1%	48	11.4%	19	4.5%
How sure are you that you could get help for yourself if you thought you were having a heart attack?	247	58.8%	108	25.7%	50	11.9%	15	3.6%

We found that 46% of participants were unable to identify heart attack signs and symptoms in someone else. 45.7% in themselves. Moreover, 60.2% of participants were unsure whether they can distinguish the signs and symptoms of a heart attack and other diseases. In addition, 61.0% were unsure if they can get help if they thought they are experiencing a heart attack, while 58.8% were unsure if they could get help for themselves.

Table 6 shows the relationship between demographic factors and the level of knowledge among the participants.

**Table 6:** The relationship between demographic factors and the level of knowledge among the participants

		Knowledge							
		High		Low		RR	CI lower	CI higher	P-value
		Count	N %	Count	N %				
Age	<40	114	42.2%	156	57.8%	Reference			
	>40	79	46.5%	91	53.5%	0.84	0.57	1.23	0.38
Gender	Male	64	35.2%	118	64.8%	1.84	1.24	2.72	0.002*
	Female	129	50.0%	129	50.0%	Reference			
Marital status	Single	62	42.8%	83	57.2%	Reference			
	Married	126	45.2%	153	54.8%	0.91	0.61	1.36	0.636
	Divorced	5	31.3%	11	68.8%	1.64	0.54	4.97	0.372
Education	Less than secondary school	4	28.6%	10	71.4%	Reference			
	Secondary school	35	42.7%	47	57.3%	0.53	0.15	1.85	0.327
	Bachelor	123	43.3%	161	56.7%	0.52	0.16	1.71	0.288
	Master	26	53.1%	23	46.9%	0.35	0.09	1.28	0.113
	Doctorate	5	45.5%	6	54.5%	0.48	0.09	2.52	0.385
Nationality	Saudi	171	42.9%	228	57.1%	Reference			
	Non-Saudi	22	53.7%	19	46.3%	0.64	0.33	1.23	0.187

We found that the age of the participants had no significant impact on their level of knowledge about the symptoms and risk factors of heart attack; however, older participants had a lower risk of low knowledge (RR = 0.84, 95% CI: 0.57:1.23, P = 0.38). Furthermore, males were more likely than females to have low knowledge (RR: 1.84, 95% CI: 1.24:2.72, P = 0.002). Other demographic factors did not effect the level of knowledge of heart attack; however, we found that higher education is associated with better knowledge, but not significantly so.

## Discussion

In this study, we looked at people’s knowledge, attitudes, and beliefs about ACS in Riyadh, Saudi Arabia. The detailed analysis of the knowledge of the participants of the symptoms of heart attack reveals that most participants recognize that chest pain is the most common symptom of ACS, followed by weakness, fatigue, and shortness of breath. However, many fail to recognize certain symptoms, such as abdominal pain, heartburn, and indigestion. Our findings were consistent with the literature findings regarding the most common symptoms of ACS [18,19], possibly because people often recognize chest pain and discomfort as classic ACS symptoms. In addition, many people are unaware that heart disease can manifest as gastrointestinal symptoms. According to an in-hospital study, most patients recognize the symptoms of ACS from a previous history or by knowing someone who has had a heart attack. However, it also demonstrated that having a personal history of ACS does not guarantee to see all of the relevant signs and may have an impact on their medical care [15]. As a result, failure to educate the community and hospitalized patients about ACS symptoms may contribute to a lack of knowledge.

In this study, obesity was the most commonly known risk factor for cardiovascular disease, followed by smoking, cholesterol, and hypertension, with 47.7% of participants having diabetes. In a previous study conducted by Alwakeel A et al., smoking was the most common risk factor identified by participants among the general population in the Tabuk region of Saudi Arabia [20]. Also, Alwakeel A et al. reported that most patients fail to recognize the relation between diabetes mellitus and ACS [20], which is consistent with our findings. It is worth noting that obesity and smoking are modifiable risk factors; thus, raising public awareness about the risk of MI associated with smoking and obesity may reduce the incidence of MI. In contrast, this study suggests that many important heart attack risk factors, including diabetes, are poorly understood. Many previous studies had similar findings [21,22].

Similar to other studies, the attitudes of our participants indicate that they would be unable to identify ACS symptoms in another person [18,23]. In addition, only 14.8% of our population believed they could help themselves in the event of a heart attack. Furthermore, the average total attitude score was similar to that reported by Dracup et al. [24] but lower than that reported by O'Brien et al. [18]. These differences can be attributed to cultural differences.

In this study, we discovered that 56% of the participants had limited knowledge of the symptoms and risk factors of MI, with no difference between participants based on educational level. Even among the well-educated participants, the Tabuk study found a lack of knowledge about the symptoms of acute myocardial infarction [20]. This can cause delays in receiving medical advice and increase mortality rates. Similar results were reported in the Kuwaiti study [21]. Among the general population in this study, the low level of knowledge of MI symptoms may be due to inadequate educational programs, a lack of knowledge of health sciences, and a small number of community health centers.

In our study, we found significant differences between men and women. Higher knowledge is found in women, which is consistent with other studies [24,25]. However, some studies oppose this finding and showed no difference in the knowledge of cardiovascular risk factors between men and women [7,20].

Limitation

The main limitation of this study is recall bias, which is a characteristic of self-administered questionnaire studies. Other limitations include the fact that we gave a list of possible symptoms of heart attack to the participants; some of them may have gotten the answers correctly based on their knowledge, while others may have assumed it correctly, which may have exaggerated the results. However, we did use a reliable and previously validated questionnaire.

## Conclusions

The results of this study can be regarded as a first step toward providing basic information on the level of knowledge about heart attacks and their risk factors among the general population in Riyadh, Saudi Arabia. This data can be used to develop future educational programs aimed at increasing awareness and knowledge of cardiovascular diseases, their risk factors, and preventive measures.
